# Lignan Intake and Type 2 Diabetes Incidence Among US Men and Women

**DOI:** 10.1001/jamanetworkopen.2024.26367

**Published:** 2024-08-07

**Authors:** Siyue Wang, Yang Hu, Binkai Liu, Yanping Li, Molin Wang, Qi Sun

**Affiliations:** 1 Department of Nutrition, Harvard T.H. Chan School of Public Health, Boston, Massachusetts; 2 Department of Epidemiology and Biostatistics, School of Public Health, Peking University, Beijing, China; 3 Department of Epidemiology, Harvard T.H. Chan School of Public Health, Boston, Massachusetts; 4 Channing Division of Network Medicine, Department of Medicine, Brigham and Women’s Hospital and Harvard Medical School, Boston, Massachusetts; 5 Department of Biostatistics, Harvard T.H. Chan School of Public Health, Boston, Massachusetts; 6 Joslin Diabetes Center, Boston, Massachusetts

## Abstract

**Importance:**

Lignans are phytoestrogens abundant in Western diets and may be associated with type 2 diabetes (T2D) risk.

**Objective:**

To prospectively investigate associations between lignan intake and T2D incidence.

**Design, Setting, and Participants:**

Population-based cohort study of US men and women enrolled in the Nurses’ Health Study (NHS, 1984-2018), NHSII (1991-2019), and Health Professionals Follow-Up Study (HPFS, 1986-2020), as well as 496 participants from the Men’s Lifestyle Validation Study (MLVS). Participants were free of T2D, cardiovascular disease, and cancer at baseline. Data were analyzed from November 2022 to July 2023.

**Exposures:**

Total and individual lignans were assessed using a validated food frequency questionnaire, which was updated every 2 to 4 years. In the MLVS, lignan intake was measured using 2 sets of 7-day diet records (7DDRs).

**Main Outcomes and Measures:**

Incident T2D cases were confirmed using American Diabetes Association diagnostic criteria. Cox proportional hazards models were used to assess multivariable-adjusted associations.

**Results:**

The current study included 201 111 participants (mean [SD] age, 44.7 [10.1] years; 161 169 female participants [80.2%]; 2614 African American participants [1.3%], 1609 Asian participants [0.8%], 2414 Hispanic and other race or ethnicity participants [1.2%], and 194 474 White participants [96.7%]) from the HPFS, NHS, and NHSII studies. The median (IQR) total lignan intake of the highest quintile ranged from 355.1 (330.2-396.9) μg/d in NHS to 459.9 (422.2-519.5) μg/d in HPFS at the median follow-up time. Over 5 068 689 person-years, 20 291 incident cases of T2D were identified. Higher lignan intake was inversely associated with T2D incidence, except for lariciresinol. The multivariable-adjusted pooled hazard ratios (HRs) for the highest vs lowest quintiles were 0.87 (95% CI, 0.83-0.91) for total lignans, 0.72 (95% CI, 0.69-0.76) for secoisolariciresinol, 0.92 (95% CI, 0.87-0.96) for pinoresinol, 0.93 (95% CI, 0.89-0.98) for matairesinol, and 0.99 (95% CI, 0.94-1.04) for lariciresinol. Secoisolariciresinol intake exhibited a significant inverse association with T2D risk among individuals with obesity (HR, 0.75 for body mass index [BMI] ≥30; 95% CI, 0.71-0.79 vs HR, 0.82 for BMI <25; 95% CI, 0.81-0.83; *P* < .001 for interaction) and premenopausal women (HR, 0.67 for premenopausal women; 95% CI, 0.65-0.69 vs HR, 0.82 for the past use of hormones; 95% CI, 0.76-0.88; *P* = .003 for interaction). Dietary lignan assessed with 7DDRs was associated with lower HbA_1c_ levels (percentage change range from −0.92% to 1.50%), as well as lower C-reactive protein levels and better lipid profiles.

**Conclusions and Relevance:**

This cohort study found that long-term lignan consumption was associated with a lower T2D risk, particularly among individuals with obesity and premenopausal women.

## Key Points

**Question:**

What is the association between lignan intake and type 2 diabetes (T2D) incidence?

**Findings:**

This cohort study of 201 111 US men and women with 5 068 689 person-years of follow-up found that higher intakes of total and individual lignans, except for lariciresinol, were associated with a lower T2D risk; among individual lignans, the inverse associations for secoisolariciresinol were more pronounced among participants with obesity, as well as among premenopausal women. In a validation study, lignan intake assessed using a 7-day diet record was also associated with lower HbA_1c_ levels and other metabolic risk markers.

**Meaning:**

These findings suggest individual plant lignans are beneficial factors for lowering T2D risk, suggesting the importance of incorporating lignin-rich foods into diets for the prevention of T2D, including flaxseed products, whole grains, and coffee.

## Teaser

This cohort study evaluates associations between lignan intake and type 2 diabetes incidence in US men and women.

## Introduction

Lignans are polyphenolic chemicals abundant in plant-based foods, such as seeds, whole grains, and certain fruits and vegetables.^1^ With estrogenic properties, lignans constitute the main dietary source of phytoestrogens in Western dietary patterns.^2^ The 4 primary plant lignans, including secoisolariciresinol, matairesinol, pinoresinol, and lariciresinol, can be metabolized to enterolignans by gut microbiota and thus are prebiotics.^3,4,5^ Enterolignans are important gut microbiota-derived metabolites and are associated with a lower risk of cardiometabolic conditions, including type 2 diabetes (T2D),^4,5,6^ heart disease,^7^ and excess weight gain.^8^ Thus far, research into the association between lignan consumption and the risk of T2D is still sparse and primarily focused on total lignan intake, with inconsistent findings. For example, a study^9^ involving 6547 Iranian adults observed an inverse association between total lignan consumption and T2D incidence, consistent with findings from the Prevención con Dieta Mediterránea (PREDIMED) trial.^10^ In contrast, null results were found in the European Prospective Investigation into Cancer (EPIC)–InterAct study.^11^ The discrepancies in the findings could be attributed to the absence of repeated lignan intake measurements, less extensive follow-up intervals, and modest sample sizes. Notably, these 4 individual lignans may have diverse conversion efficiencies in the human body after being absorbed and metabolized,^12^ and hence may exhibit different patterns of associations with several cardiometabolic traits.^1,13^ However, we know of no study to date that has assessed the association between intakes of these 4 individual lignans and T2D incidence.^14,15^

To fill this knowledge gap, with repeated measurements across 3 large, prospective US cohorts with over 30 years of follow-up, we aimed to assess the association between total lignan intake and 4 individual lignan intakes and T2D risk. In addition, we further explored whether body weight or postmenopausal status or hormone use might modify the associations of interest, and also examined associations between lignan consumption, plasma enterolactone levels, and T2D risk markers, including glycated hemoglobin (HbA_1c_), C-reactive protein (CRP), and lipid profiles in a small substudy.

## Methods

The current study was approved by the institutional review boards at the Harvard T.H. Chan School of Public Health and Brigham and Women’s Hospital, with returned questionnaires constituting implied consent. This study followed the Strengthening the Reporting of Observational Studies in Epidemiology (STROBE) reporting guideline.

### Study Population

The Nurses’ Health Study (NHS), initiated in 1976, enrolled 121 700 female nurses aged 30 to 55 years who completed a questionnaire on medical, lifestyle, and health-related information. In 1989, the Nurses’ Health Study II (NHSII) was launched, enrolling 116 340 female nurses aged 25 to 42 years. Parallel to the female cohorts, the Health Professionals Follow-up Study (HPFS) began in 1986, recruiting 51 529 US male health professionals aged 40 to 75 years. Baseline data for all cohorts were collected using validated questionnaires, with biennial follow-ups to update anthropometric, lifestyle, medical history, and other characteristics. The average cumulative response rate across all cohorts was at least 90%.

Diet was first assessed in 1984 for NHS, 1991 for NHSII, and 1986 for HPFS using a validated semiquantitative food frequency questionnaire (FFQ). Follow-up data were collected through 2018 for NHS, 2019 for NHSII, and 2020 for HPFS. Participants with prevalent T2D, cardiovascular disease, or cancer at baseline; missing age or demographic data; implausible energy intake (<500 or >3500 kcal/d for women, <800 or >4200 kcal/d for men); incomplete baseline questionnaires; or missing lignan intake data were excluded.

### Men’s Lifestyle Validation Study

We examined associations between lignan intake assessed using 7-day diet records (7DDRs), plasma enterolactone levels, and HbA_1c_ in the Men’s Lifestyle Valiation Study (MLVS). In brief, the MLVS,^16^ conducted between 2012 and 2013, is a substudy involving male participants from the HPFS and the Harvard Pilgrim Health Care study. Its primary objective was to evaluate the validity of self-reported dietary and lifestyle data. The current analysis was conducted among 496 participants from the MLVS-HPFS cohort who had complete 7DDRs for lignan intake, available blood samples for metabolic risk markers, and valid enterolactone measurements.

### Assessment of Lignan Intake

Total and individual lignan intakes were calculated by multiplying the consumption frequency of lignan-containing foods with their respective lignan contents, which are from the Harvard University Food Composition Database and the US department of agriculture (USDA).^17^ The lignan-containing foods were assessed with a validated FFQ, updated every 2 to 4 years since baseline. The FFQ detailed approximately 130 items, recording yearly consumption frequency and portion sizes with 9 response options, from “never or less than once a month” to “6+ per day”. The calculated values for each food item were aggregated to determine the overall and individual lignan intakes. Notably, as the intake levels and primary contributors of individual lignans may change over time, we identified the top 10 contributing foods for each type of lignan consumed throughout the follow-up period in the cohorts.^1^ It is worth mentioning that flaxseed has been added to the FFQ since 2006 to 2007, which leads to a significant increase in mean secoisolariciresinol intake levels observed since 2006 to 2007. Our studies^1^ have shown that FFQ-assessed lignan intake was significantly correlated with that measured using 7DDRs (*r* = 0.53; *P* < .001) and that lignan intake was also significantly associated with plasma enterolactone concentrations (*r* = 0.30; *P* < .001) in the MLVS.

In the MLVS, diet was assessed using 2 sets of 7DDRs. Briefly, participants recorded the preconsumption and postconsumption weights of all foods in grams to determine actual intake, using standardized food scales and rulers, for 7 consecutive days. Additionally, participants provided recipes for home-prepared meals with serving sizes and amount consumed. Dietary information was then integrated with the Nutrition Data System for Research 2011 database^18^ to ascertain the intakes of total and 4 individual lignans.

### Demographic and Lifestyle Assessments

Demographic and lifestyle factors, along with health outcomes, were updated every 2 years through comprehensive questionnaires. Such data included race, body weight, smoking habits, alcohol intake, and multivitamin supplements. Race was assessed in this study to account for potential demographic diversity of the study population (Asian, African American, Hispanic and other races and ethnicities, and White). For women, menopausal status and postmenopausal hormone use were also inquired. Validation studies showed that self-reported weight was highly correlated with measured weights (*r* = 0.96).^19^ Recreational physical activity was assessed through a validated questionnaire^20^ that inquired about the average time devoted to engaging in 10 commonly performed activities. To quantify the energy expenditure associated with these activities, we computed the weekly energy expenditure in metabolic equivalent hours. Diet quality was assessed using a modified Alternate Healthy Eating Index (AHEI) without alcohol, for which nuts and legumes, whole grains, and vegetables that are among the major sources of lignans were further removed.^21^ The modified AHEI summarized the intake levels of the remaining 7 items, including fruits, sugar-sweetened beverages and fruit juice, red and processed meat, trans-fat, long chain n-3 fats, polyunsaturated fats, and sodium.

### Ascertainment of T2D

Participants who reported a physician’s diagnosis of diabetes underwent further verification through a supplementary diagnostic questionnaire that inquired about diagnostic tests, symptoms, and hypoglycemic therapy. A case of diabetes was confirmed if it met any of these criteria: the presence of classic symptoms along with a fasting plasma glucose level of 126 mg/dL or higher, a random plasma glucose level of 200 mg/dL or higher (to convert glucose to micromoles per liter, multiply by .0555); multiple instances of elevated plasma glucose levels on separate occasions without symptoms; or if the individual was receiving hypoglycemic medication. Our validation studies demonstrated a high level of validity, with 98% of questionnaire-confirmed diabetes cases in the NHS and 97% in the HPFS being reconfirmed through medical record review.

### Assessment of Enterolactone and Other Biomarkers in the MLVS

In the MLVS, fasting blood samples were collected biannually and were processed to isolate the plasma. Plasma enterolactone concentrations were assessed using electrospray ionization orbitrap liquid chromatography mass spectrometry (model Q-Exactive, Thermo Scientific, Inc). The methods for assessing enterolactone and diabetes-related biomarkers, including HbA_1c_, high-density lipoprotein cholesterol (HDL-C) and triglycerides (TG), have been detailed in previous publications.^5,22^

### Statistical Analysis

To account for the upward trend in lignan intake observed during the follow-up, the characteristics of the participants were presented at the median of follow-up (2000 for NHS and HPFS, 2001 for NHSII). In order to reflect long-term habitual intake levels, both total and individual lignan intake were cumulatively averaged. To mitigate potential reverse causality, where participants with new diagnoses might modify their diets, we stopped updating the diet once participants developed stroke or cancer or underwent coronary artery bypass graft during the follow-up period.

The accumulation of person-time for follow-up began from the return date of the baseline questionnaire for each participant and concluded at the earliest of the following events: diagnosis of T2D, death, the return of the last valid follow-up questionnaire, or the end of the follow-up (June 30, 2018, in NHS; June 30, 2019, in NHSII; and January 31, 2020, in HPFS). A multivariable Cox proportional hazards model, stratified by age and calendar time, was used to calculate the hazard ratios (HRs) and their 95% CIs, assessing the association between total and individual lignan intake and the risk of T2D. We tested the proportional hazards assumption by adding a multiplicative interaction of lignan by follow-up duration and found no violations. Total and individual lignan intake were categorized into quintiles. Covariates considered in the multivariable models included race and ethnicity, total energy intake (quintiles), baseline body mass index (BMI; calculated as weight in kilograms divided by height in meters squared), total energy intake (quintiles), smoking habits, alcohol intake, multivitamin use (yes or no), physical activity (quintiles), hypertension (yes or no), hypercholesterinemia (yes or no), family history of diabetes (yes or no), postmenopausal hormone use (women only), modified AHEI (quintiles), and oral contraceptive use (yes or no; women only). To minimize missing values of covariates, a valid value was carried forward to replace missing data at the next cycle, and we then used a missing indicator variable to address the remaining missing values. The median value of total and individual lignan intakes within each quintile was modeled as a continuous variable to estimate HRs, 95% CIs, and *P* for trend. Initial analyses were conducted within each cohort, after which the HRs were pooled using an inverse variance-weighted method. Heterogeneity among cohorts was evaluated using the Cochran Q test.

In the MLVS, the association between lignan intake and HbA_1c_ was assessed using the multivariable linear regression models. We used averaged intake of lignans determined from 2 sets of 7DDR measurements. Covariates in multivariable analyses included age at blood draw, physical activity, BMI at blood draw, alcohol intake, smoking, total energy intake, and healthy plant-based diet index derived from 7DDRs. The lignan intake and HbA_1c_, along with other biomarkers, were log-transformed, standardized to *z* scores, and assessed for Gaussian normality to meet the assumptions of the linear regression model. Statistical estimates are presented as percentage changes in HbA_1c_ and their 95% CIs for each 10 μg/d increase in lignan intake. The percentage differences with corresponding 95% CIs were calculated by back-transforming effect estimates using 1 + ([1 + 10%]^β^ − 1) × 100%. We also examined the role of enterolactone levels in associations between lignan intake and cardiometabolic risk markers.

Based on prior knowledge and hypothesis,^23,24^ we tested for potential effect modification by baseline BMI (<25, 25-29.9, and ≥30), and premenopausal or any use of postmenopausal hormones (never hormone use, past hormone use, or current hormone use). We assessed the interactions by incorporating a cross-product term of the categorical variables and tertiled lignan intake in the full models, and the significance of interaction was determined with the log-likelihood ratio test. We further presented HRs by a joint association analysis, and observed the trend in each group of effect modifiers, adjusting for the same covariates as in the primary analysis. Cohorts were pooled for the interaction and joint association analyses.

To examine whether the association between lignan intake and T2D incidence was mediated by time-varying BMI, a mediation test was performed using the %MEDIATE SAS macro.^25^ We quantified the extent to which time-varying BMI explained the association by comparing the strength of the association between lignan intake and T2D both before and after accounting for the mediator, which was computed as follows:

(1 − [*β*_1 _/ *β*_2_]) × 100%

where β_2_ represents the regression coefficient of dietary lignans without adjustment of the mediator, and β_1_ represents the coefficient after adjustments. In a sensitivity analysis, we assessed the association independent of flaxseed consumption by repeating the analysis after excluding participants who indicated flaxseed consumption throughout the follow-up period.

All statistical tests were conducted using SAS version 9.3 (SAS Institute) and were 2-sided with a significance level of .05. Data analysis was conducted between November 2022 and July 2023.

## Results

A total of 201 111 participants (mean [SD] age, 44.7 [10.1] years; 161 169 [80.2%] female participants) were included at baseline. Participants included 2614 (1.3%) African American participants, 1609 (0.8%) Asian participants, 2414 (1.2%) Hispanic and other race or ethnicity participants, and 194 474 (96.7%) White participants. Table 1 shows the age-standardized demographic and lifestyle features of study participants in NHS, NHSII, and HPFS by lignan intake quintiles at the median follow-up. The median (IQR) total lignan intake of the highest quintiles ranged from 355.1 (330.2-396.9) μg/d in NHS to 459.9 (422.2-519.5) μg/d in HPFS at the median follow-up time. Individuals with higher total lignan intake tended to be older and exhibited healthier characteristics, such as lower BMI, higher overall dietary quality, greater physical activity, and lower prevalence of hypertension and hypercholesterolemia.

**Table 1.** Age-Standardized Characteristics of Study Participants in Nurses’ Health Study (NHS), Nurses’ Health Study II (NHSII), and Health Professionals Follow-up Study (HPFS) at Median Follow-Up^a^

Study and characteristic	Participants by total lignan intake, No. (%)				
	Q1	Q2	Q3	Q4	Q5
NHS (2000)					
No. of participants	12 271	12 271	12 272	12 271	12 271
Lignans, median (IQR), μg/d					
Total	176.1 (158.5-188.5)	216.1 (207.8-224.4)	248.6 (240.4-257.2)	286.2 (275.8-298.6)	355.1 (330.2-396.9)
Matairesinol	5.4 (4.4-6.6)	6.9 (5.7-8.6)	8.3 (6.6-10.6)	10 (7.7-13.3)	14.1 (9.8-20)
Secoisolariciresinol	61.1 (51.6-70.4)	74.9 (66.2-84.2)	83.5 (74.5-93.5)	91.5 (81.2-102.6)	104.5 (91.5-119.9)
Pinoresinol	37 (31.4-42.9)	47.5 (41.7-53.6)	55.9 (49.6-62.8)	66.5 (59-74.5)	88 (76.3-104.1)
Lariciresinol	66.8 (58.7-74.8)	84.9 (77.5-92.2)	99 (90.8-107.4)	116.3 (106.3-126.8)	149.8 (133.1-171.6)
Age, mean (SD), y^b^	62.7 (7.2)	63.6 (7.1)	64 (7.0)	64.6 (6.9)	65.6 (6.9)
Body mass index, mean (SD)^c^	24.8 (4.3)	24.7 (4.1)	24.5 (4.0)	24.3 (3.8)	24.0 (3.7)
Race and ethnicity					
African American	86 (0.7)	74 (0.6)	74 (0.6)	74 (0.6)	98 (0.8)
Asian	184 (1.5)	147 (1.2)	98 (0.8)	122 (1.0)	147 (1.2)
Hispanic and other	25 (0.2)	25 (0.2)	25 (0.2)	37 (0.3)	37 (0.3)
White	11 976 (97.6)	12 025 (98.0)	12 075 (98.3)	12 038 (98.1)	11 989 (97.7)
Physical activity, median (IQR), MET-h/wk	7.6 (2.3-17.1)	9.3 (3.2-20.5)	10.9 (4.2-23.1)	13.1 (4.8-26.2)	15.9 (6.3-32.1)
Hypertension	3890 (31.7)	3706 (30.2)	3657 (29.8)	3571 (29.1)	3411 (27.8)
High cholesterol	4086 (33.3)	4013 (32.7)	4074 (33.2)	4123 (33.6)	4000 (32.6)
Smoking status					
Never smokers	5927 (48.3)	5682 (46.3)	5471 (44.5)	5203 (42.4)	5166 (42.1)
Past smokers	4737 (38.6)	5227 (42.6)	5608 (45.6)	5878 (47.9)	6025 (49.1)
Current smokers	1607 (13.1)	1362 (11.1)	1203 (9.8)	1190 (9.7)	1080 (8.8)
Family history of diabetes	3289 (26.8)	3301 (26.9)	3387 (27.6)	3362 (27.4)	3302 (26.9)
Multivitamin use	6295 (51.3)	6847 (55.8)	7265 (59.2)	7503 (61.1)	7945 (64.7)
Oral contraceptive ever use	6160 (50.2)	6111 (49.8)	6259 (51.0)	6366 (51.8)	6280 (51.1)
Hormone use					
Premenopausal	810 (6.6)	798 (6.5)	736 (6.0)	761 (6.2)	724 (5.9)
Postmenopausal, never	3289 (26.8)	3042 (24.9)	2896 (23.6)	2786 (22.7)	2835 (23.1)
Postmenopausal, current	5448 (44.4)	5780 (47.1)	5989 (48.7)	6160 (50.2)	6025 (49.1)
Postmenopausal, past	2724 (22.2)	2651 (21.6)	2651 (21.6)	2664 (20.9)	2687 (21.9)
Alcohol consumption, median (IQR), g/d	1.0 (0.0-5.0)	2.0 (0.3-7.0)	2.5 (0.5-8.0)	3.1 (0.6-9.3)	3.1 (0.6-9.6)
Modified Alternative Healthy Eating Index, median (IQR)^d^	30.9 (26.8-35.3)	33.1 (29.0-37.4)	34.7 (30.6-39.1)	36.5 (32.4-40.8)	39.7 (35.2-44.3)
Total energy intake, median (IQR), Kcal/d	1711 (1430-2034)	1723 (1449-2023)	1718 (1444-2031)	1698 (1432-2013)	1702 (1423-2007)
NHS II (2001)					
No. of participants	16 676	16 675	16 676	16 676	16 675
Lignans, median (IQR), μg/d					
Total	163.6 (142.6-178.3)	211.6 (201.5-221.3)	250.7 (240.7-261.2)	296.8 (283.8-311.8)	383.4 (351.3-446.7)
Matairesinol	5.6 (4.5-7.3)	8 (6.2-10.1)	9.8 (7.6-12.6)	12.2 (9.4-16.1)	17.3 (12.4-24.1)
Secoisolariciresinol	49.1 (40.1-58.9)	66.6 (57.4-76.3)	77.5 (67.2-88.3)	88.2 (76.9-100.0)	107.6 (92.6-127.7)
Pinoresinol	37.4 (31.5-43.4)	49.7 (43.8-56.0)	59.9 (53.0-67.0)	72.4 (64.6-81.0)	96.4 (83.8-113.4)
Lariciresinol	64.7 (55.5-73.0)	85.2 (77.6-93.0)	101.8 (93.0-110.7)	122.4 (111.8-133.3)	161.2 (143.1-187.0)
Age, mean (SD), y^b^	47.5 (4.7)	48.3 (4.6)	48.7 (4.5)	49.2 (4.5)	49.8 (4.4)
Body mass index, mean (SD)^c^	24.8 (5.2)	24.4 (4.7)	24.2 (4.5)	24.0 (4.4)	23.5 (4.1)
Race					
White	15 992 (95.9)	16 104 (96.6)	16 176 (97.1)	16 182 (97.1)	16 075 (96.4)
African American	67 (0.4)	83 (0.5)	82 (0.5)	98 (0.6)	117 (0.7)
Asian	267 (1.6)	250 (1.5)	234 (1.4)	213 (1.3)	283 (1.7)
Hispanic and other	350 (2.1)	233 (1.4)	184 (1.1)	183 (1.1)	200 (1.2)
Physical activity, median (IQR), MET-h/wk	7.7 (2.3-18.5)	10.0 (3.5-22.4)	12.1 (4.3-26.1)	15.2 (5.9-30.9)	20.3 (8.2-39.4)
Hypertension	22.7	21.8	20.4	18.9	17.6
High cholesterol	36.1	34.0	33.0	32.7	31.5
Smoking status					
Never smokers	12 107 (72.6)	11 306 (67.8)	10 789 (64.7)	10 339 (62.0)	10 188 (61.1)
Past smokers	3102 (18.6)	3919 (23.5)	4469 (26.8)	4936 (29.6)	5253 (31.5)
Current smokers	1467 (8.8)	1451 (8.7)	1418 (8.5)	1401 (8.4)	1234 (7.4)
Family history of diabetes	7204 (43.2)	6953 (41.7)	6987 (41.9)	6887 (41.3)	6820 (40.9)
Multivitamin use	8638 (51.8)	9471 (56.8)	9990 (59.9)	10 406 (62.4)	10 922 (65.5)
Oral contraceptive ever use	15 042 (90.2)	15 108 (90.6)	15 209 (91.2)	15 192 (91.1)	15 174 (91.0)
Hormone use					
Premenopausal	9822 (58.9)	9872 (59.2)	9956 (59.7)	9906 (59.4)	9922 (59.5)
Postmenopausal, never	1551 (9.3)	1467 (8.8)	1417 (8.5)	1467 (8.8)	1434 (8.6)
Postmenopausal, current	2818 (16.9)	2802 (16.8)	2768 (16.6)	2735 (16.5)	2718 (16.3)
Postmenopausal, past	2485 (14.9)	2535 (15.2)	2535 (15.2)	2568 (15.4)	2601 (15.5)
Alcohol consumption, median (IQR), (g/d)	0.5 (0.0-2.1)	1.2 (0.0-4.0)	1.8 (0.3-5.4)	2.4 (0.5-6.8)	2.6 (0.5-7.3)
Modified Alternative Healthy Eating Index, median (IQR)^d^	29.1 (25-33.7)	31.5 (27.3-35.9)	33.4 (29.2-37.9)	35.6 (31.5-39.9)	39.3 (34.8-43.8)
Total energy intake, median (IQR), Kcal/d	1759 (1449-2113)	1788 (1486-2130)	1783 (1484-2128)	1773 (1480-2111)	1766 (1473-2107)
HPFS (2000)					
No. of participants	6274	6274	6275	6274	6274
Lignans, median (IQR), μg/d					
Total	197.2 (173-213.4)	252.8 (241.3-264.2)	299.1 (286.9-311.6)	354.3 (338.7-372.5)	459.9 (422.2-519.5)
Matairesinol	6.2 (4.9-7.8)	8.0 (6.3-10.4)	9.8 (7.4-13.2)	12.2 (8.8-18.0)	18.7 (11.1-29.6)
Secoisolariciresinol	64.1 (53.3-76.2)	81.9 (70.8-94.2)	92.2 (79.8-105)	102.5 (88.7-117.6)	117.0 (99.4-139.1)
Pinoresinol	42.6 (34.8-50.2)	58.5 (50.8-67.1)	72.1 (63.0-82.5)	89.1 (77.3-102.5)	125.7 (105.5-158.6)
Lariciresinol	76.3 (65.2-86.3)	100.7 (91.4-110.3)	120.8 (110.1-132.4)	145.3 (131.7-159.7)	193.2 (169.3-223.4)
Age, mean (SD), y^b^	64.2 (8.8)	65.0 (8.9)	65.0 (8.7)	65.5 (8.7)	66.1 (8.6)
Body mass index, mean (SD)^c^	25.3 (2.8)	25.3 (2.7)	25.2 (2.6)	25.1 (2.6)	24.7 (2.6)
Race					
African American	151 (2.4)	113 (1.8)	138 (2.2)	125 (2.0)	144 (2.3)
Asian	100 (1.7)	88 (1.4)	101 (1.6)	103 (1.6)	87 (1.4)
Hispanic and other	75 (1.2)	31 (0.5)	50 (0.8)	36 (0.5)	51 (0.8)
White	5948 (94.8)	6042 (96.3)	5986 (95.4)	6010 (95.8)	5992 (95.5)
Physical activity, median (IQR), MET-h/wk	15.9 (4.8-36.6)	20.5 (7.4-41.4)	22.2 (8.3-43.5)	25.4 (9.9-48.1)	27.7 (10.9-52.7)
Hypertension	2428 (38.7)	2453 (39.1)	2378 (37.9)	2397 (38.2)	2372 (37.8)
High cholesterol	3012 (48.0)	3062 (48.8)	3138 (50.0)	3118 (49.7)	3099 (49.4)
Smoking status					
Never smokers	3313 (52.9)	2905 (46.3)	2918 (46.5)	2811 (44.8)	2736 (43.6)
Past smokers	2541 (40.5)	2999 (47.8)	3056 (48.7)	3218 (51.3)	3269 (52.1)
Current smokers	420 (6.7)	370 (5.8)	301 (4.8)	245 (3.9)	269 (4.3)
Family history of MI	1311 (20.9)	1387 (22.1)	1355 (21.6)	1343 (21.4)	1318 (21.0)
Multivitamin use	3087 (49.2)	3375 (53.8)	3602 (57.4)	3764 (60.0)	3821 (60.9)
Alcohol consumption, median (IQR), g/d	2.3 (0-8.1.0)	5.5 (1.2-13.1)	7.6 (2.0-15.3)	9.0 (2.9-17.5)	10.5 (3.0-23.3)
Modified Alternative Healthy Eating Index, median (IQR)^d^	30.4 (25.7-35.7)	32.8 (28.1-37.9)	34.4 (29.7-39.5)	36.7 (31.7-41.7)	39.5 (34.4-44.2)
Total energy intake, median (IQR), Kcal/d	1936 (1583-2342)	1935 (1609-2317)	1938 (1604-2327)	1925 (1592-2303)	1920 (1597-2287)

Abbreviations: MET-h/wk, metabolic equivalent of task hours per week; MI, myocardial infarction; Q, quintile.

^a^ Values are standardized to the age distribution of the study population. Values of polytomous variables may not sum to 100% due to rounding.

^b^ Value is not age adjusted.

^c^ Body mass index is calculated as weight in kilograms divided by height in meters squared.

^d^ Modified Alternative Healthy Eating Index (AHEI) removes indices from nuts, whole grains, and vegetables from the total AHEI score (no alcohol).

Table 2 presents the main associations between lignan intake and T2D risk across the 3 cohorts. During 5 068 689 person-years of follow-up, 20 291 T2D cases were documented. The overall mean (SD) lignan intake level across the full follow-up period of 3 cohorts was 371.8 (1167.5) μg/d. Higher intakes of total and individual lignans, except for lariciresinol, were associated with approximately 8% to 27% lower T2D incidence (approximate HR, 0.72-0.93). Comparing the highest with the lowest quintiles of intake, the multivariable adjusted pooled HRs for T2D were 0.87 (95% CI, 0.83-0.91) for total lignans (*P* for trend < .001), 0.72 (95% CI, 0.69-0.76) for secoisolariciresinol (*P* for trend < .001), 0.92 (95% CI, 0.87-0.96) for pinoresinol (*P* for trend < .001), 0.93 (95% CI, 0.89-0.98) for matairesinol (*P* for trend = .01), and 0.99 (95% CI, 0.94-1.04) for lariciresinol (*P* for trend = .46). The association was generally homogenous across the 3 cohorts.

**Table 2.** Associations Between Lignans Intake and Type 2 Diabetes Risk in Nurses’ Health Study (NHS; 1984-2018), Nurses’ Health Study II (NHSII; 1991-2019), and Health Professionals Follow-up Study (HPFS; 1986-2020)

Lignan	Participants by lignan intake, hazard ratio (95% CI)^a^					*P* value for trend^b^
	Q1	Q2	Q3	Q4	Q5	
Total lignans	170.1 (163.7177.9)	216.2 (207.3226.5)	253.9 (246.3266.4)	299.3 (292.9314.7)	390.2 (381.6422.3)	NA
NHS						
Cases, No./person-year	2077/378 144	1846/378 414	1620/378 594	1464/378 883	1238/379 594	NA
Age-adjusted	1 [Reference]	0.88 (0.82-0.93)	0.76 (0.71-0.81)	0.68 (0.64-0.73)	0.57 (0.53-0.61)	<.001
Multivariable-adjusted^c^	1 [Reference]	1.00 (0.93-1.06)	0.93 (0.87-0.99)	0.91 (0.85-0.98)	0.87 (0.80-0.94)	<.001
NHS II						
Cases, No./person-year	2104/442 389	1714/442 990	1594/443 621	1409/443 771	1139/443 782	NA
Age-adjusted	1 [Reference]	0.78 (0.73-0.83)	0.69 (0.65-0.74)	0.59 (0.55-0.64)	0.47 (0.43-0.50)	<.001
Multivariable-adjusted^c^	1 [Reference]	0.96 (0.90-1.02)	0.98 (0.91-1.05)	0.93 (0.87-1.01)	0.87 (0.80-0.95)	.002
HPFS						
Cases, No./person-year	1007/191 249	943/191 368	816/191 703	708/191 981	612/192 206	NA
Age-adjusted	1 [Reference]	0.92 (0.84-1.01)	0.79 (0.72-0.87)	0.68 (0.62-0.75)	0.58 (0.53-0.64)	<.001
Multivariable-adjusted^c^	1 [Reference]	1.01 (0.92-1.11)	0.94 (0.85-1.03)	0.88 (0.79-0.98)	0.87 (0.78-0.97)	.002
Pooled						
Cases, No./person-year	5188/1 011 782	4503/1 012 772	4030/1 013 918	3581/1 014 635	2989/1 015 582	NA
Age-adjusted	1 [Reference]	0.84 (0.81- 0.88)	0.74 (0.71- 0.77)	0.65 (0.62- 0.67)	0.53 (0.50- 0.55)	<.001
Multivariable-adjusted^c^	1 [Reference]	0.98 (0.95- 1.03)	0.95 (0.91- 0.99)	0.91 (0.87- 0.96)	0.87 (0.83- 0.91)	<.001
*P* for heterogeneity^d^	NA	.58	.52	.64	.99	NA
Secoisolariciresinol	53.3 (49.8-67.4)	71.3 (64.9-82.5)	82.0 (70.6-94.5)	93.8 (80.6116.1)	116.4 (94.4118.6)	NA
NHS						
Cases, No./person-year	2360/378 013	1830/378 591	1633/378 607	1411/379 007	1011/379 412	NA
Age-adjusted	1 [Reference]	0.76 (0.71-0.80)	0.67 (0.63-0.72)	0.58 (0.54-0.62)	0.41 (0.38-0.44)	<.001
Multivariable-adjusted^c^	1 [Reference]	0.86 (0.81-0.92)	0.87 (0.82-0.93)	0.81 (0.76-0.87)	0.66 (0.61-0.71)	<.001
NHS II						
Cases, No./person-year	2277/442 067	1845/442 899	1524/443 611	1270/444 119	1044/443 857	NA
Age-adjusted	1 [Reference]	0.76 (0.72-0.81)	0.60 (0.56-0.64)	0.48 (0.45-0.51)	0.38 (0.35-0.41)	<.001
Multivariable-adjusted^c^	1 [Reference]	0.95 (0.89-1.01)	0.87 (0.82-0.94)	0.80 (0.74-0.86)	0.75 (0.69-0.82)	.001
HPFS						
Cases, No./person-year	983/191 213	879/191 674	873/191 518	709/192 021	642/192 081	NA
Age-adjusted	1 [Reference]	0.87 (0.80-0.96)	0.86 (0.78-0.94)	0.69 (0.63-0.76)	0.62 (0.56-0.68)	<.001
Multivariable-adjusted^c^	1 [Reference]	0.93 (0.85-1.02)	0.96 (0.87-1.05)	0.80 (0.73-0.89)	0.78 (0.70-0.87)	<.001
Pooled						
Cases, No./person-year	5620/1 011 293	4554/1 013 164	4030/1 013 736	3390/1 015 147	2697/1 015 350	NA
Age-adjusted	1 [Reference]	0.78 (0.75- 0.81)	0.68 (0.65- 0.71)	0.56 (0.53- 0.58)	0.43 (0.41- 0.45)	<.001
Multivariable-adjusted^c^	1 [Reference]	0.91 (0.87- 0.95)	0.89 (0.85- 0.93)	0.80 (0.77- 0.84)	0.72 (0.69- 0.76)	<.001
*P* for heterogeneity^d^	NA	.07	.25	.94	.02	NA
Matairesinol	5.6 (4.6-7.2)	7.5 (5.6-9.8)	8.7 (6.6-11.5)	10.7 (7.8-16.2)	14.7 (10.0-20.1)	NA
NHS						
Cases, No./person-year	1842/377 599	1717/378 377	1684/378 549	1612/379 382	1390/379 722	NA
Age-adjusted	1 [Reference]	0.92 (0.86-0.99)	0.90 (0.84-0.96)	0.85 (0.80-0.91)	0.72 (0.68-0.78)	<.001
Multivariable-adjusted^c^	1 [Reference]	0.97 (0.91-1.04)	0.97 (0.91-1.04)	0.94 (0.88-1.01)	0.91 (0.84-0.98)	0.04
NHS II						
Cases, No./person-year	1743/442 708	1681/443 663	1557/443 445	1566/443 458	1413/443 279	NA
Age-adjusted	1 [Reference]	0.93 (0.87-1.00)	0.86 (0.80-0.92)	0.85 (0.79-0.91)	0.74 (0.69-0.80)	<.001
Multivariable-adjusted^c^	1 [Reference]	1.02 (0.95-1.09)	0.99 (0.92-1.06)	1.04 (0.97-1.12)	0.99 (0.92-1.06)	.81
HPFS						
Cases, No./person-year	895/190 735	895/191 586	814/191 543	802/192 017	680/192 625	NA
Age-adjusted	1 [Reference]	0.98 (0.90-1.08)	0.89 (0.81-0.98)	0.86 (0.78-0.95)	0.72 (0.65-0.80)	<.001
Multivariable-adjusted^c^	1 [Reference]	0.97 (0.89-1.07)	0.92 (0.84-1.01)	0.94 (0.85-1.04)	0.87 (0.78-0.97)	.02
Pooled						
Cases, No./person-year	4480/1 011 042	4293/1 013 626	4055/1 013 537	3980/1 014 857	3483/1 015 626	NA
Age-adjusted	1 [Reference]	0.94 (0.90-0.98)	0.88 (0.84-0.92)	0.85 (0.82-0.89)	0.73 (0.70-0.76)	<.001
Multivariable-adjusted^c^	1 [Reference]	0.99 (0.95-1.03)	0.97 (0.93-1.01)	0.98 (0.94-1.03)	0.93 (0.89-0.98)	.01
*P* for heterogeneity^d^	NA	.54	.50	.09	.12	NA
Pinoresinol	37.5 (33.6-43.5)	49.6 (42.0-57.5)	59.0 (52.0-70.1)	71.6 (61.9-82.7)	99.5 (80.9103.8)	NA
NHS						
Cases, No./person-year	1918/378 058	1798/378 394	1672/378 638	1605/378 725	1252/379 814	NA
Age-adjusted	1 [Reference]	0.94 (0.88-1.00)	0.86 (0.80-0.92)	0.82 (0.76-0.87)	0.64 (0.59-0.68)	<.001
Multivariable-adjusted^c^	1 [Reference]	0.99 (0.93-1.06)	0.95 (0.89-1.02)	0.96 (0.90-1.03)	0.89 (0.82-0.96)	.003
NHS II						
Cases, No./person-year	1944/442 457	1706/443 340	1656/443 291	1451/443 735	1203/443 730	NA
Age-adjusted	1 [Reference]	0.86 (0.80-0.92)	0.82 (0.76-0.87)	0.70 (0.66-0.75)	0.57 (0.53-0.61)	<.001
Multivariable-adjusted^c^	1 [Reference]	1.01 (0.94-1.08)	1.04 (0.97-1.12)	1.01 (0.94-1.08)	0.99 (0.92-1.08)	.82
HPFS						
Cases, No./person-year	1005/191 185	970/191 431	796/191 749	751/191 987	564/192 155	NA
Age-adjusted	1 [Reference]	0.96 (0.88-1.05)	0.78 (0.71-0.86)	0.73 (0.67-0.81)	0.55 (0.50-0.61)	<.001
Multivariable-adjusted^c^	1 [Reference]	1.04 (0.95-1.13)	0.92 (0.83-1.01)	0.94 (0.85-1.04)	0.84 (0.75-0.94)	.001
Pooled						
Cases, No./person-year	4867/1 011 700	4474/1 013 165	4124/1 013 678	3807/1 014 447	3019/1 015 699	NA
Age-adjusted	1 [Reference]	0.91 (0.87-0.95)	0.83 (0.79-0.86)	0.75 (0.72-0.79)	0.59 (0.56-0.62)	<.001
Multivariable-adjusted^b^	1 [Reference]	1.01 (0.97-1.05)	0.98 (0.94-1.02)	0.98 (0.93-1.02)	0.92 (0.87-0.96)	<.001
*P* for heterogeneity^d^	NA	.71	.06	.49	.03	NA
Lariciresinol	65.9 (63.1-72.1)	85.1 (81.2-98.2)	102.5 (93.4120.0)	123.6 (108.7130.0)	165.1 (164.5166.2)	NA
NHS						
Cases, No./person-year	1854/378 500	1773/378 594	1613/378 735	1582/378 702	1423/379 098	NA
Age-adjusted	1 [Reference]	0.95 (0.89-1.02)	0.86 (0.80-0.92)	0.83 (0.78-0.89)	0.74 (0.69-0.79)	<.001
Multivariable-adjusted^c^	1 [Reference]	1.05 (0.99-1.13)	1.01 (0.94-1.08)	1.01 (0.94-1.09)	1.02 (0.95-1.11)	.84
NHS II						
Cases, No./person-year	1956/442 994	1644/443 454	1582/443 312	1482/443 396	1296/443 397	NA
Age-adjusted	1 [Reference]	0.82 (0.76-0.87)	0.76 (0.71-0.82)	0.69 (0.65-0.74)	0.59 (0.55-0.63)	<.001
Multivariable-adjusted^c^	1 [Reference]	1.00 (0.94-1.07)	1.02 (0.95-1.10)	1.01 (0.93-1.08)	1.01 (0.93-1.09)	.90
HPFS						
Cases, No./person-year	997/191 251	882/191 616	811/191 744	747/191 906	649/191 989	NA
Age-adjusted	1 [Reference]	0.87 (0.80-0.96)	0.80 (0.73-0.88)	0.73 (0.67-0.81)	0.63 (0.57-0.70)	<.001
Multivariable-adjusted^c^	1 [Reference]	0.95 (0.86-1.04)	0.95 (0.86-1.04)	0.92 (0.84-1.02)	0.91 (0.81-1.01)	.09
Pooled						
Cases, No./person-year	4807/1 012 745	4299/1 013 664	4006/1 013 791	3811/1 014 004	3368/1 014 484	NA
Age-adjusted	1 [Reference]	0.88 (0.85-0.92)	0.81 (0.77-0.84)	0.75 (0.72-0.79)	0.65 (0.63-0.68)	<.001
Multivariable-adjusted^b^	1 [Reference]	1.01 (0.97-1.05)	1.00 (0.96-1.05)	0.99 (0.95-1.04)	0.99 (0.94-1.04)	.46
*P* for heterogeneity^d^	NA	.18	.40	.31	.19	NA

Abbreviations: NA, not applicable; Q, quintile.

^a^ Median value and IQR in each Q were calculated across the full follow-up period of 3 cohorts.

^b^ *P* value for trend was calculated using median values in each Q as the continuous exposure in the model.

^c^ Hazard ratios were meta-analyzed using fixed effect models. Models were age-stratified (months) and calendar-time–stratified and adjusted for race and ethnicity (African American, Asian, Hispanic and other, or White), total energy intake (Qs), smoking status (never smoked, past smoker, currently smoke 1-14 cigarettes per day, 15-24 cigarettes per day, or ≥25 cigarettes per day), baseline BMI (calculated as weight in kilograms divided by height in meters squared; <21.0, 21.0-22.9, 23.0-24.9, 25.0-26.9, 27.0-29.9, 30.0-32.9, 33.0-34.9, or ≥35.0), alcohol intake (0, 0.1-4.9, 5.0-9.9, 10.0-14.9, 15.0-29.9, or ≥30.0 g/d), multivitamin use (yes or no), physical activity (Qs), modified Alternative Healthy Index removing indices of nuts, whole grains, and vegetables, and family history of diabetes. For women, postmenopausal hormone use (premenopausal, never, former, current, or missing), and oral contraceptive ever use were further adjusted.

^d^ *P* for heterogeneity was measured by Q statistic with 1 degree of freedom.

Of individual lignans, secoisolariciresinol (but not other lignans) was more greatly associated with lower T2D risk among participants with baseline BMI over 30 (HR, 0.75 for BMI ≥30; 95% CI, 0.71-0.79 vs HR, 0.82 for BMI <25; 95% CI, 0.81-0.83; *P *for interaction < .001), as well as the group of premenopausal women (HR, 0.67 for the premenopausal group; 95% CI, 0.65-0.69 vs HR, 0.82 for the past use of hormones group; 95% CI, 0.76-0.88; *P *for interaction = .003). The Figure presents the joint association between lignan intake and BMI in all 3 cohorts, and the eFigure in Supplement 1 presents the association between lignan intake and menopause and hormone use in the female cohorts (NHS and NHSII).

**Figure.**
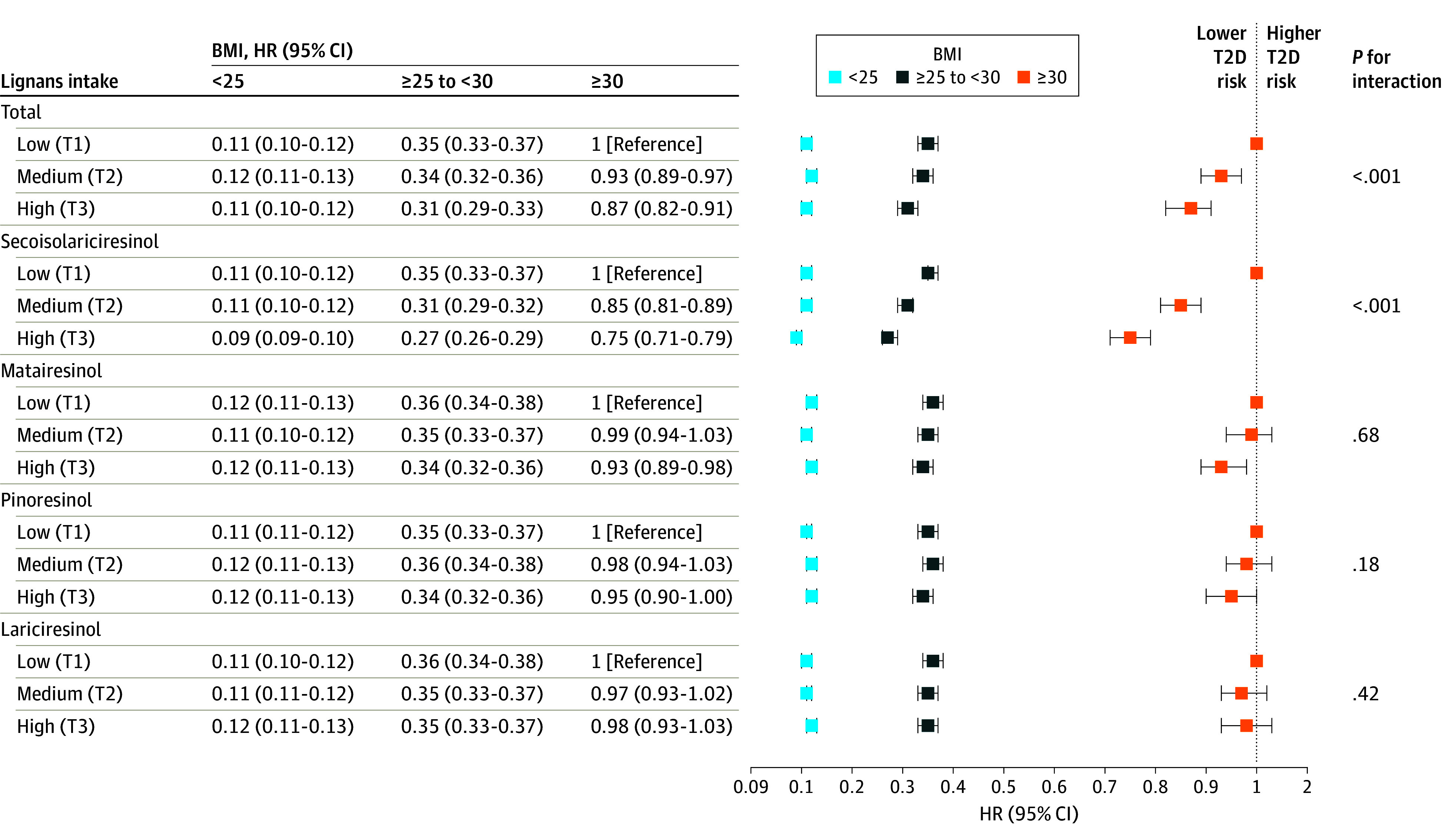
Joint Association Between Lignans Intake and Body Mass Index (BMI) in Association With Incident Type 2 Diabetes (T2D) Risk The baseline BMI (calculated as weight in kilograms divided by height in meters squared) group was categorized as low (<25), medium (25≤ to <30) and high (≥30) and was analyzed from the 3 pooled cohorts; total and individual lignan intakes were categorized with their respective tertile (T) values. Models were age-stratified (months) and calendar-time–stratified and adjusted for ethnicity (African American, Asian, Hispanic and other, or White), total energy intake (quintiles); smoking status (never smoked, past smoker, currently smoke 1-14 cigarettes per day, 15-24 cigarettes per day, or ≥25 cigarettes per day); alcohol intake (0, 0.1-4.9, 5.0-9.9, 10.0-14.9, 15.0-29.9, or ≥30.0 g/d); multivitamin use (yes or no); physical activity (quintiles); modified Alternative Healthy Index removing indices of nuts, whole grains, and vegetables; and family history of diabetes. For women, oral contraceptive ever use was further adjusted for the BMI analysis only. *P* value for interaction was calculated from the likelihood ratio test.

In the MLVS, 7DDR-assessed lignan intake was significantly associated with lower HbA_1c_ levels (eTable 1 in Supplement 1). The percentage change of HbA_1c_ *z* score corresponding to a 10% change in intake *z* score was −1.33% (95% CI, −2.18% to −0.47%) for total lignan (*P *= .003), −1.05% (95% CI, −1.90% to −0.20%) for secoisolariciresinol (*P* = .02), −1.28% (95% CI, −2.13% to −0.38%) for matairesinol (*P* = .01), −1.30% (95% CI, −2.18% to −0.40%) for pinoresinol (*P* = .01), and −1.58% (95% CI, −2.48% to −0.68%) for lariciresinol (*P* < .001). With further adjustments for enterolactone levels, the percentage change of HbA_1c_ *z* score per 10% change in total lignan intake *z* score was slightly attenuated to −1.23% (95% CI, −2.15% to −0.30%). The 4 individual lignans assessed with 7DDRs were also associated with lower HbA_1c_ levels independent of plasma enterolactone levels, with percentage changes ranging from −0.92% to −1.50%. eTable 2 in Supplement 1 shows that higher plasma enterolactone levels were associated with lower HbA_1c_ (*r*_s_ = −0.10; *P* = .02), lower CRP (*r*_s_ = −0.18; *P* < .001), and better lipid profiles, characterized by lower TG (*r*_s_ = −0.15; *P* = .001), and higher HDL-C (*r*_s_ = 0.14; *P* = .002).

Results from the mediation analyses showed that time-varying BMI may partially mediate the association between total and individual lignan intake and T2D incidence. Specifically, for each SD increase per μg/d in total lignan intake, time-varying BMI accounted for 31.3% (95% CI, 22.7%-41.4%; *P* < .001) of the association after adjusting for all the covariates listed in Table 2. Of individual lignans, time-varying BMI accounted for 33.2% (95% CI, 28.6%-38.1%; *P* < .001) of the association for secoisolariciresinol, 25.2% (95% CI, 10.8%-48.3; *P* < .001) of the association for matairesinol, and 41.7% (95% CI, 21.5%-65.2%; *P* < .001) of the association for pinoresinol. In the sensitivity analysis that excluded participants consuming flaxseeds, we found a similar association with the main analysis, where robust association was observed between total and individual lignans, with the exception of lariciresinol, in association with T2D risk (eTable 3 in Supplement 1).

## Discussion

In the current study, higher intakes of total and individual lignans, except for lariciresinol, were associated with approximately 8% to 27% lower T2D incidence (approximate HR, 0.72-0.93). Of individual lignans, the inverse associations for secoisolariciresinol intakes were more pronounced among participants with obesity, as well as among premenopausal women. Dietary lignan intake assessed using 7DDRs was also associated with lower HbA_1c_ levels. These associations were independent of established and potential confounders and robust in sensitivity analyses.

In the Tehran Lipid and Glucose Study^9^ consisting of 6547 Iranian adults with an average of 3 years follow-up, total lignan intake was associated with a strikingly lower T2D risk (HR comparing extreme tertiles: 0.60; 95% CI, 0.43-0.85). In the PREDIMED study,^24^ a similar association was observed: comparing extreme lignan intake tertiles, the HR was 0.71 (95% CI, 0.51-0.98). Our observation of total lignan intake in association with T2D risk was in line with these studies, although the effect size was smaller, probably because of relatively lower lignan intake levels (mean: 371.8 μg/d) in our study participants in comparison with those in the Iranian^9^ (approximately 3.6-5.6 mg/d) and Spanish^24^ (approximately 1.5-1.6 mg/d) studies. Despite similar magnitude with our observed associations, the inverse association (HR, 0.88; 95% CI, 0.72-1.07) between lignan intake and T2D incidence did not achieve significance at the EPIC-InterAct case-cohort study.^11^ This inconsistency between the current and existing studies underscores the complexity of establishing a clear dose-response association across a wide range of lignan intakes, and may also be partially explained by differences in study design and statistical power on the ability to detect modest associations. It remains unknown whether the beneficial effects of lignans on T2D risk may plateau at certain intake levels. More studies with adequate power to explore the full spectrum of lignan intake and its relationship with T2D risk are needed to substantiate the associations.

Our study generated novel evidence for individual plant lignans in association with T2D risk. Of individual lignans, we observed the greatest inverse association for secoisolariciresinol, while lariciresinol intake was not associated with T2D. This differentiation in associations could possibly be attributed to variations in the bioavailability of individual lignans in the human body,^26^ where differences in individual lignan chemical structures,^12^ ability in binding enzymes,^27^ and interactions with other compounds^13^ are possible reasons, thus leading to different patterns of relationships with T2D. In vitro^28^ and animal studies^29^ indicated that secoisolariciresinol may exhibit higher bioavailability than lariciresinol under specific conditions. This could potentially support our findings that intake of secoisolariciresinol, but not lariciresinol, was associated with T2D risk. However, more studies are warranted to fully elucidate the differences among individual lignans. It is also important to consider that the dietary sources and overall dietary patterns associated with higher intakes of specific lignans may introduce confounding factors that could influence the observed associations. For example, individuals who consume more secoisolariciresinol may also have diets that are richer in flaxseeds, whole grain cereals, and tea,^21,30^ which are known to be beneficial for glycemic control. However, we have controlled for AHEI to address confounding by diet quality. Nevertheless, our findings regarding secoisolariciresinol are in line with existing evidence generated from clinical trials that focused on glycemic traits. Several recent meta-analyses of randomized clinical trials demonstrated that among individuals with prediabetes and T2D, flaxseed supplementation significantly reduced levels of fasting blood glucose, insulin, HbA_1c_, and HOMA-IR.^31,32^ This is consistent with findings from another meta-analysis among healthy individuals showing that lignan intake contributed to better glycemic control.^33^ The correlation is further supported by our findings in the MLVS, where a higher intake of lignans was associated with decreased levels of HbA_1c_. Notably, most clinical trials used secoisolariciresinol diglucoside (SDG), a precursor of secoisolariciresinol, to evaluate the health effects of lignan consumption, possibly due to its bioavailability and the human body’s ability to convert SDG into secoisolariciresinol.^26,30^ However, the association between SDG and glucose metabolism remains inconsistent. Specifically, Eriksen et al^34^ reported no improvement in glucose metabolism despite administering a high dose of SDG. The variability in individual responses to lignan supplementation, as evidenced by the meta-analysis of flaxseed supplementation trials,^32^ underscores the importance of considering dosage, duration, and individual variability when assessing the health benefits of lignans. Taken together, existing evidence supports the notion that lignan intake, in particular secoisolariciresinol, may contribute to T2D prevention or glycemic control, although, again, more studies are warranted to substantiate our findings.

In our analyses, we observed greater inverse associations between secoisolariciresinol intake and T2D risk among participants with obesity and premenopausal women. In a recent Japanese cohort study,^35^ similar inverse associations were noted between isoflavone consumption and serum lipid profiles among individuals with overweight and obesity as compared with the normal weight and lean group. In contrast, a cross-sectional study^24^ reported a greater inverse association of polyphenol intake with T2D prevalence among the group with overweight as compared with the group with obesity. Regarding menopausal status, a study of US women found that urinary excretion of isoflavones was linked to lower T2D risk, particularly among postmenopausal women not using hormone therapy.^36^ Potential reasons underlying these discrepancies is unknown. It is likely that body weight may influence the accuracy of dietary measurements,^37^ but given the prospective study design, such measurement errors are largely nondifferential and attenuate true associations of interest. In the MLVS, the associations between FFQ-assessed lignan intake, 7DDR-assessed lignan intake, and plasma enterolactone concentrations were generally consistent across BMI groups, suggesting that BMI was not associated with the measurement errors. Further studies are needed to replicate the associations that depend on BMI or menopausal status.^36^

The mechanisms underlying the potential benefits of lignans in controlling the T2D risks are not yet fully elucidated. In vitro and in vivo experimental studies have indicated that lignans possess antidiabetic properties, including enhancements in insulin sensitivity, regulation of glucose homeostasis, obesity prevention, and protection against oxidative stress and inflammation—all of which are closely linked to the development of T2D.^3,12,13,27,38,39,40^ Our previous work^4,5^ has further emphasized the significance of plant lignan conversion to enterolactone by the gut microbiota. The complex interrelationships among plant lignan intake, microbial composition, and enterolignans in the context of T2D risk reduction requires further exploration. Concerning the effect modification by obesity status, it is plausible that lignans’ beneficial effects on reducing oxidative stress and alleviating low-grade inflammation may be particularly effective in diabetes prevention among individuals with obesity who typically have an adverse profile of systemic inflammation, oxidative stress, and insulin resistance.^41^ Furthermore, previous studies supported the notion that the more apparent benefits of lignan consumption among individuals with obesity could be attributed to their potential role in managing body weight.^8,42^ The observed greater inverse association among premenopausal women might be attributed to the close relationship between lignans and sex hormone metabolism.^43^ Lignans can exhibit either estrogenic or antiestrogenic effects depending on the individual’s endogenous estrogen levels^44^ and also tissue.^26,30^ Typically, premenopausal women have higher endogenous estrogen levels, leading lignans to predominantly exert antiestrogenic effects.^45^ These antiestrogenic actions elevate the levels of sex hormone-binding proteins,^46^ which are significantly associated with a reduced risk of T2D.^47^ In support of this concept, a recent review^43^ has highlighted that the effects of phytoestrogens, like lignans, are contingent upon various factors, including the hormonal status of the individual consumer.

### Strengths and Limitations

This study has several notable strengths. First, by repeatedly measuring lignan intake, we minimized within-person measurement error while capturing dynamic changes in intake over time. Second, we comprehensively investigate lignan intake across individual types. Lastly, our study benefits from a large population size and an extensive follow-up period spanning over 30 years.

Several limitations merit discussion. First, flaxseed intake was not assessed during early follow-up of the cohorts, which might lead to misclassification of secoisolariciresinol intake levels during early follow-up. However, it is important to note that flaxseed consumption remained relatively low throughout the study. Moreover, a sensitivity analysis excluding participants with flaxseed consumption still demonstrated a robust association between secoisolariciresinol intake and T2D incidence. Second, despite comprehensive and repeated assessments of lignans, measurement errors are inevitable. However, as discussed previously, given the prospective study design, the measurement errors are more likely to be nondifferential and may bias true associations toward the null. Third, despite multivariable adjustments, we could not fully rule out the possibility that unmeasured or residual confounding may partially explain the observed associations. Additionally, the socioeconomic and racial homogeneity of our study populations may facilitate better control for confounding by these characteristics, although the generalizability of our findings is limited. As such, further investigations among populations with more diverse socioeconomic and racial backgrounds are warranted.

## Conclusions

In summary, findings from 3 large prospective US cohorts suggest that higher total lignan intake as well as higher secoisolariciresinol, matairesinol, and pinoresinol (but not lariciresinol) were significantly associated with a reduced T2D risk. The inverse associations between secoisolariciresinol intake and T2D risk appeared to be more pronounced among participants with obesity or premenopausal women. Our findings underscore the importance of a healthy plant-based diet rich in lignan-containing foods, including flaxseed products, whole grains, and coffee for the primary prevention of T2D.

## Notes

Supplement 1. **eFigure.** Joint Association Between Lignan Intake and Menopause and Hormone Use Status in Association With Incident Type 2 Diabetes (T2D) Risk **eTable 1.** The Association Between Dietary Lignans Intake and HbA_1c_% Level in Men’s Lifestyle Validation Study (MLVS) **eTable 2.** Association Between Plasma Enterolactone Level and Diabetes-Related Biomarkers in Men’s Lifestyle Validation Study (MLVS) **eTable 3.** Pooled Associations Between Lignans Intake and Type 2 Diabetes Risk Excluding Participants Consuming Flaxseeds

Supplement 2.
Data Sharing Statement
