# Supplementary material for: Lignan Intake and Type 2 Diabetes Incidence Among US Men and Women
Source: JAMA Netw Open. 2024 Aug 7;7(8):e2426367. doi: 10.1001/jamanetworkopen.2024.26367 (PMC11307137; doi:10.1001/jamanetworkopen.2024.26367)
Supplement: Supplement 1. — eFigure. Joint Association Between Lignan Intake and Menopause and Hormone Use Status in Association With Incident Type 2 Diabetes (T2D) Risk eTable 1. The Association Between Dietary Lignans Intake and HbA1c% Level in Men’s Lifestyle Validation Study (MLVS) eTable 2. Association Between Plasma Enterolactone Level and Diabetes-Related Biomarkers in Men’s Lifestyle Validation Study (MLVS) eTable 3. Pooled Associations Between Lignans Intake and Type 2 Diabetes Risk Excluding Participants Consuming Flaxseeds [file jamanetwopen-e2426367-s001.pdf]

Supplemental Online Content

Wang S, Hu Y, Liu B, Li Y, Wang M, Sun Q. Lignan intake and type 2 diabetes incidence among US men and women. *JAMA Netw Open*. 2024;7(8):e2426367. doi:10.1001/jamanetworkopen.2024.26367

eFigure. Joint Association Between Lignan Intake and Menopause and Hormone Use Status in Association With Incident Type 2 Diabetes (T2D) Risk
eTable 1. The Association Between Dietary Lignans Intake and HbA_{1c}% Level in Men's Lifestyle Validation Study (MLVS)
eTable 2. Association Between Plasma Enterolactone Level and Diabetes-Related Biomarkers in Men's Lifestyle Validation Study (MLVS)
eTable 3. Pooled Associations Between Lignans Intake and Type 2 Diabetes Risk Excluding Participants Consuming Flaxseeds

This supplemental material has been provided by the authors to give readers additional information about their work.

© 2024 Wang S et al. *JAMA Network Open.*

eFigure. Joint Association Between Lignan Intake and Menopause and Hormone Use Status in Association With Incident Type 2 Diabetes (T2D) Risk

■ Premenopause ■ HormoneNeverUse ■ HormoneCurrentUse ■ HormonePastUse

Lignans Intake	HR(95%CI) Premenopause	HormoneNeverUse	HormoneCurrentUse	HormonePastUse	P for interaction
**Total**					
Low (T1)	0.78(0.72,0.86)	0.98(0.91,1.05)	0.77(0.71,0.83)	1.00(Reference)	
Medium (T2)	0.73(0.66,0.80)	0.99(0.91,1.06)	0.73(0.67,0.79)	0.95(0.89,1.02)	0.107
High (T3)	0.65(0.59,0.73)	0.82(0.75,0.90)	0.71(0.65,0.78)	0.93(0.86,1.01)	
**Seco**					
Low (T1)	0.84(0.77,0.92)	1.00(0.93,1.07)	0.80(0.74,0.87)	1.00(Reference)	
Medium (T2)	0.64(0.58,0.71)	0.91(0.84,0.98)	0.69(0.64,0.75)	0.93(0.88,1.00)	0.003
High (T3)	0.56(0.50,0.63)	0.76(0.70,0.84)	0.61(0.55,0.67)	0.82(0.76,0.88)	
**Mat**					
Low (T1)	0.77(0.70,0.84)	0.97(0.90,1.04)	0.70(0.64,0.79)	1.00(Reference)	
Medium (T2)	0.72(0.65,0.79)	0.97(0.90,1.05)	0.78(0.72,0.85)	0.96(0.90,1.03)	0.061
High (T3)	0.72(0.65,0.80)	0.88(0.81,0.96)	0.75(0.68,0.81)	0.95(0.88,1.02)	
**Pino**					
Low (T1)	0.77(0.70,0.84)	1.01(0.94,1.09)	0.77(0.71,0.83)	1.00(Reference)	
Medium (T2)	0.78(0.71,0.86)	0.97(0.90,1.05)	0.77(0.71,0.83)	1.01(0.94,1.08)	0.629
High (T3)	0.71(0.64,0.79)	0.91(0.83,0.99)	0.77(0.70,0.84)	0.98(0.90,1.06)	
**Laric**					
Low (T1)	0.77(0.70,0.85)	0.97(0.90,1.04)	0.76(0.70,0.83)	1.00(Reference)	
Medium (T2)	0.74(0.67,0.82)	1.01(0.94,1.10)	0.76(0.70,0.82)	1.01(0.94,1.08)	0.521
High (T3)	0.78(0.70,0.87)	0.96(0.88,1.04)	0.81(0.74,0.89)	1.01(0.94,1.10)	

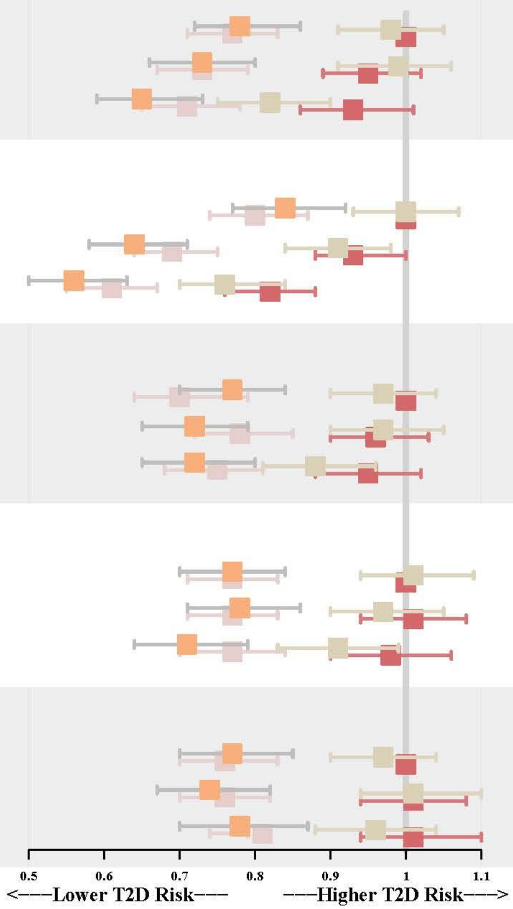

Data were analyzed within Nurses' Health Study (NHS) and Nurses' Health Study II (NHSII), which were female cohorts. Menopause and hormone use status were investigated in the NHS and NHSII only, and were grouped as the premenopausal group, hormone never use group, hormone current use group, and hormone past use group; total and individual lignan intakes were categorized with their respective tertile (T) values. Models were age-

© 2024 Wang S et al. *JAMA Network Open.*

stratified (months) and calendar-time–stratified and adjusted for race and ethnicity (African American, Asian, Hispanic and other, or White); total energy intake (quintiles); smoking status (never smoked, past smoker, currently smoke 1-14 cigarettes per day, 15-24 cigarettes per day, or ≥25 cigarettes per day); baseline BMI (calculated as weight in kilograms divided by height in meters squared; <21.0, 21.0-22.9, 23.0-24.9, 25.0-26.9, 27.0-29.9, 30.0-32.9, 33.0-34.9, or ≥35.0); alcohol intake (0, 0.1-4.9, 5.0-9.9, 10.0-14.9, 15.0-29.9, or ≥30.0 g/d); multivitamin use (yes or no); physical activity (quintiles); modified Alternative Healthy Index removing indices of nuts, whole grains, and vegetables; and family history of diabetes. *P* value for interaction was calculated from the likelihood ratio test.

© 2024 Wang S et al. *JAMA Network Open.*

eTable 1. The Association Between Dietary Lignans Intake and HbA_{1c}% Level in Men's Lifestyle Validation Study (MLVS)

Lignan Type		Percent Change (95% CI)[c]	P value
Total lignans			
	Model 1[a]	-1.33%(-2.18%,-0.47%)	0.003
	Model 2[b]	-1.23%(-2.15%,-0.30%)	0.01
Secoisolariciresinol			
	Model 1[a]	-1.05%(-1.90%,-0.20%)	0.02
	Model 2[b]	-0.92%(-1.82%,-0.01%)	0.05
Matairesinol			
	Model 1[a]	-1.28%(-2.13%,-0.38%)	0.006
	Model 2[b]	-1.15%(-2.05%,-0.24%)	0.01
Pinoresinol			
	Model 1[a]	-1.30%(-2.18%,-0.40%)	0.005
	Model 2[b]	-1.18%(-2.10%,-0.26%)	0.01
Lariciresinol			
	Model 1[a]	-1.58%(-2.48%,-0.68%)	<0.001
	Model 2[b]	-1.50%(-2.44%,-0.54%)	0.002

[a] Model 1 adjusted for age at blood draw, 7DDR smoking status (never smoked, past smoker, currently smoke), 7DDR physical activity (quintiles), 7DDR healthy plant-based diet index (quintiles), 7DDR alcohol intake (quintiles) and 7DDR total energy intake (Kcal/day).

[b] Model 2 additionally adjusted for plasma enterolactone level.

[c] Percent change was the averaged percent change in HbA1c% corresponding to 10% change in lignans intake. Both HbA1c% and lignans intake were log-transformed and standardized with per SD. The percent change of HbA1c% corresponding to 10% change in lignans intake=$(1.10^{\beta}-1)*100\%$; where β is the parameter estimate from the linear regression model.

© 2024 Wang S et al. *JAMA Network Open.*

eTable 2. Association Between Plasma Enterolactone Level and Diabetes-Related Biomarkers in Men's Lifestyle Validation Study (MLVS)

Biomarker[a]	Spearman Correlation	P for spearman	Percent Change[b], 95%CI	P for linear regression[c]
HbA1c%	-0.10	0.02	-0.72%(-1.58%,0.15%)	0.10
CRP	-0.18	<.001	-1.27%(-2.13%,-0.41%)	0.004
TG	-0.15	0.001	-0.90%(-1.76%,-0.03%)	0.04
HDL_c	0.14	0.002	0.74%(-0.20%,1.69%)	0.12

[a] HbA1c%: glycated hemoglobin percentage, CRP: c-reactive protein, TG: Triglyceride, HDL_c: high-density lipoprotein cholesterol

[b] Percent change was the averaged percent change in biomarkers corresponding to 10% change in enterolactone level. Both biomarkers and enterolactone level were log-transformed and standardized with per SD. The percent change of biomarker corresponding to 10% change in enterolactone level=$(1.10^{\beta}-1)*100\%$; where β is the parameter estimate from the linear regression model.

[c] Models were adjusted for age at blood draw, 7DDR smoking status (never smoked, past smoker, currently smoke), 7DDR physical activity (quintiles), 7DDR healthy plant-based diet index (quintiles), 7DDR alcohol intake (quintiles) and 7DDR total energy intake (Kcal/day).

© 2024 Wang S et al. *JAMA Network Open.*

eTable 3. Pooled Associations Between Lignans Intake and Type 2 Diabetes Risk Excluding Participants Consuming Flaxseeds

	Q1	Q2	Q3	Q4	Q5	*P* for trend[a]
**Total lignans**						
Case/person-year	5,177/1,009,740	4,501/1,010,756	4,027/1,011,862	3,571/1,012,619	2,987/1,013,507	
Age-adjusted	1.00 (Reference)	0.85 (0.81, 0.88)	0.74 (0.71, 0.77)	0.64 (0.62, 0.67)	0.53 (0.51, 0.55)	<.001
Multivariable-adjusted[b]	1.00 (Reference)	0.99 (0.95, 1.03)	0.95 (0.91, 0.99)	0.91 (0.87, 0.96)	0.87 (0.83, 0.92)	<.001
P for heterogeneity	--	0.52	0.53	0.59	0.98	
**Secoisolariciresinol**						
Case/person-year	5,611/1,009,272	4,554/1,011,106	4,020/1,011,707	3,379/1,013,133	2,699/1,013,265	
Age-adjusted	1.00 (Reference)	0.78 (0.75, 0.81)	0.68 (0.65, 0.71)	0.55 (0.53, 0.58)	0.43 (0.41, 0.46)	<.001
Multivariable-adjusted[b]	1.00 (Reference)	0.91 (0.88, 0.95)	0.89 (0.85, 0.93)	0.80 (0.77, 0.84)	0.72 (0.69, 0.76)	<.001
P for heterogeneity	--	0.06	0.27	0.93	0.01	
**Matairesinol**						
Case/person-year	4,472/1,009,010	4,286/1,011,620	4,050/1,011,459	3,979/1,012,838	3,476/1,013,556	
Age-adjusted	1.00 (Reference)	0.94 (0.90, 0.98)	0.88 (0.85, 0.92)	0.85 (0.82, 0.89)	0.73 (0.70, 0.76)	<.001
Multivariable-adjusted[b]	1.00 (Reference)	0.99 (0.95, 1.03)	0.97 (0.93, 1.01)	0.98 (0.94, 1.03)	0.93 (0.89, 0.98)	0.01
P for heterogeneity	--	0.55	0.54	0.10	0.11	
**Pinoresinol**						
Case/person-year	4,859/1,009,676	4,465/1,011,139	4,126/1,011,628	3,795/1,012,403	3,018/1,013,636	
Age-adjusted	1.00 (Reference)	0.91 (0.87, 0.95)	0.83 (0.79, 0.86)	0.75 (0.72, 0.79)	0.59 (0.56, 0.62)	<.001
Multivariable-adjusted[b]	1.00 (Reference)	1.01 (0.96, 1.05)	0.98 (0.94, 1.02)	0.97 (0.93, 1.02)	0.92 (0.87, 0.97)	<.001
P for heterogeneity	--	0.72	0.08	0.43	0.04	
**Lariciresinol**						
Case/person-year	4,797/1,010,726	4,297/1,011,624	4,003/1,011,742	3,798/1,011,990	3,368/1,012,400	
Age-adjusted	1.00 (Reference)	0.88 (0.85, 0.92)	0.81 (0.77, 0.84)	0.75 (0.72, 0.79)	0.66 (0.63, 0.69)	<.001
Multivariable-adjusted[b]	1.00 (Reference)	1.01 (0.97, 1.06)	1.00 (0.96, 1.05)	0.99 (0.95, 1.04)	0.99 (0.95, 1.05)	0.48
P for heterogeneity	--	0.24	0.47	0.26	0.24	

[a] Median value in each quintile was used to calculate *P* value for trend.

[b] HRs were pooled using a fixed-effects model. Model adjusted for the same covariates as listed in Table 2.

© 2024 Wang S et al. *JAMA Network Open.*
