# Supplementary material for: Lignan Intake and Type 2 Diabetes Incidence Among US Men and Women
Source: JAMA Netw Open. 2024 Aug 7;7(8):e2426367. doi: 10.1001/jamanetworkopen.2024.26367 (PMC11307137; doi:10.1001/jamanetworkopen.2024.26367)
Supplement: Supplement 2. — Data Sharing Statement [file jamanetwopen-e2426367-s002.pdf]

Data Sharing Statement

Wang. Lignan Intake and Type 2 Diabetes Incidence Among US Men and Women. *JAMA Netw Open*. Published August 07, 2024. doi:10.1001/jamanetworkopen.2024.26367

Data
Data available: Yes
Data types: Data dictionary
How to access data: Because of participant confidentiality and privacy concerns, data cannot be shared publicly and requests to access NHS/NHSII/HPFS data must be submitted in writing. According to standard controlled access procedures, applications to use NHS/NHSII/HPFS resources will be reviewed by our External Collaborations Committee to verify that the proposed use maintains the protection of the privacy of participants and the confidentiality of the data. Investigators wishing to use NHS/NHSII/HPFS data are asked to submit a brief description of the proposed project (go to https://www.nurseshealthstudy.org/researchers (contact email: nhsaccess@channing.harvard.edu) and https://sites.sph.harvard.edu/hpfs/for-collaborators/ for details.
When available: With publication

Supporting Documents
Document types: None

Additional Information
Who can access the data: Because of participant confidentiality and privacy concerns, data cannot be shared publicly and requests to access NHS/NHSII/HPFS data must be submitted in writing. According to standard controlled access procedures, applications to use NHS/NHSII/HPFS resources will be reviewed by our External Collaborations Committee to verify that the proposed use maintains the protection of the privacy of participants and the confidentiality of the data. Investigators wishing to use NHS/NHSII/HPFS data are asked to submit a brief description of the proposed project (go to https://www.nurseshealthstudy.org/researchers (contact email: nhsaccess@channing.harvard.edu) and https://sites.sph.harvard.edu/hpfs/for-collaborators/ for details.
Types of analyses: Because of participant confidentiality and privacy concerns, data cannot be shared publicly and requests to access NHS/NHSII/HPFS data must be submitted in writing. According to standard controlled access procedures, applications to use NHS/NHSII/HPFS resources will be reviewed by our External Collaborations Committee to verify that the proposed use maintains the protection of the privacy of participants and the confidentiality of the data. Investigators wishing to use NHS/NHSII/HPFS data are asked to submit a brief description of the proposed project (go to https://www.nurseshealthstudy.org/researchers (contact email: nhsaccess@channing.harvard.edu) and https://sites.sph.harvard.edu/hpfs/for-collaborators/ for details.
Mechanisms of data availability: Because of participant confidentiality and privacy concerns, data cannot be shared publicly and requests to access NHS/NHSII/HPFS data must be submitted in writing. According to standard controlled access procedures, applications to use NHS/NHSII/HPFS resources will be reviewed by our External Collaborations Committee to verify that the proposed use maintains the protection of the privacy of participants and the confidentiality of the data. Investigators wishing to use NHS/NHSII/HPFS data are asked to submit a brief description of the proposed project (go to https://www.nurseshealthstudy.org/researchers (contact email: nhsaccess@channing.harvard.edu) and https://sites.sph.harvard.edu/hpfs/for-collaborators/ for details.
